# Mobile health in the specific management of first-episode psychosis: a systematic literature review

**DOI:** 10.3389/fpsyt.2023.1137644

**Published:** 2023-06-12

**Authors:** Claire Maechling, Antoine Yrondi, Amandine Cambon

**Affiliations:** ^1^Pôle de Psychiatrie, Centre Hospitalier Universitaire de Toulouse, Toulouse, France; ^2^Service de Psychiatrie et de Psychologie Médicale, Centre Expert Dépression Résistante Fonda Mental, CHU de Toulouse, Hôpital Purpan, ToNIC Toulouse NeuroImaging Centre, Université de Toulouse, INSERM, UPS, Toulouse, France; ^3^Programme d'intervention précoce RePeps, réseau Transition, Clinique Aufrery, Toulouse, France

**Keywords:** early psychosis, first-episode psychosis, early intervention, mobile health, mobile applications, digital intervention, smartphone

## Abstract

**Purpose:**

The purpose of this systematic literature review is to assess the therapeutic efficacy of mobile health methods in the management of patients with first-episode psychosis (FEP).

**Method:**

The participants are patients with FEP. The interventions are smartphone applications. The studies assess the preliminary efficacy of various types of application.

**Results:**

One study found that monitoring symptoms minimized relapses, visits to A&E and hospital admissions, while one study showed a decrease in positive psychotic symptoms. One study found an improvement in anxiety symptoms and two studies noted an improvement in psychotic symptoms. One study demonstrated its efficacy in helping participants return to studying and employment and one study reported improved motivation.

**Conclusion:**

The studies suggest that mobile applications have potential value in the management of young patients with FEP through the use of various assessment and intervention tools. This systematic review has several limitations due to the lack of randomized controlled studies available in the literature.

## 1. Introduction

### 1.1. Early intervention in psychosis

The central issues in emerging psychosis are accessing and maintaining effective care. One factor that justifies early intervention is the stage of life at which FEP occurs: late adolescence and early adulthood. This period coincides with a critical phase of development, in which life plans can be disrupted. The aim of early intervention in psychosis (EIP) is to provide recovery-oriented care for young patients with FEP (1).

The first goal of early intervention is to reduce the duration of untreated psychosis (DUP). Long DUP may have a significant social impact, accentuating social withdrawal and stigmatization (1). The other goals concern the prevention of relapse and of unfavorable long-term evolution. The aim is to prevent allowing functional disability and social exclusion to set in (1).

The two main components of EIP are an adapted form of case management and the initiation of treatment as soon as possible after FEP (2).

The deployment of EIP programmes has been an important development in recent decades. Current scientific evidence suggests that these programmes are associated with better outcomes than standard treatment in the early stages of psychosis. A recent meta-analysis including 10 randomized controlled trials on three continents (Europe, North America and Asia) demonstrates the superiority of EIP programmes in terms of treatment discontinuation rates, proportion of patients requiring hospitalization, occupational and educational progression, global functioning and severity of positive and negative symptoms (3).

### 1.2. Patient engagement

Patient engagement in care is fragile for psychotic disorders, and FEP in particular. A Swiss study estimated that 50% of patients were lost to follow-up or disengaged after their first hospitalization in a standard psychiatric department (4). The greater the patient's engagement, the lower the risk of relapse. Early intervention programmes aim to involve patients in their care by fostering a therapeutic alliance. AS a result, they limit the traumatic nature of early psychotic experiences (1).

Nevertheless, a significant proportion of patients (20.5%−40%) still drop out of specialized follow-up within the first 2 years (5). Factors associated with disengagement from early intervention programmes are lack of family support, poor adherence to treatment, substance misuse, coming from an ethnic minority and having a criminal record (5, 6). To reduce the risk of disengagement, particular attention should be paid to these factors by offering targeted intervention. These factors at patient level may thus influence engagement, but the level of care provided may also be at the root of patient disengagement. The most common reason given by patients is that the care does not meet their needs (7).

### 1.3. mHealth

Recovery is now one of main objective in the field of schizophrenia-related disorder (mainly FEP) (8). Recovery includes, amongst others, perceived social integration and empowerment (9). However, individuals with psychosis report experiences of loneliness and social withdrawal (10, 11). The Survey of High Impact Psychosis indicates that loneliness and social withdrawal rank second on the list of challenges to recovery (12–14). Moreover, the face-to-face relationship could be reduced for individuals with psychosis due to a diminution of pleasure (anhedonia) and a sense of threat (15). Schlosser et al. (16) highlighted that internet-based interventions focusing on social connection could decrease social withdrawal in this population. Moreover, young patients going through FEP fear being stigmatized, that clinicians do not acknowledge their experiences and are unable to respond appropriately to their needs (17). Technology can offer the possibility of accessing resources or coping strategies without the fear of the stigma associated with mental health. It can provide platforms for young people to share their experiences and feel supported, provide new ways of working with their careers, allow more accurate assessment of their symptoms and promote positive changes in their daily lives. In addition, mobile health could help patient to increase their own empowerment (18).

Studies in Canada show that most young people admitted for FEP have access to a smartphone (19). A study conducted in an EIP programme in Montreal shows that over 90% of young people diagnosed with FEP have access to a smartphone (20) and many are receptive to using this technology for their mental health care (21). People with FEP report that the use of mobile technologies could be an acceptable way to access mental health information and support, decrease the stigma associated with care and could provide a sense of control over their recovery (referred to internationally as “empowerment”) (22). Most of these young people are open to using technology to receive therapy (21), to get in touch with their peers with similar problems, and they particularly appreciate it when the sites are professionally moderated (23).

The restrictions resulting from the COVID-19 pandemic have heightened the demand for mental health care and imposed a reorganization of our care system by stepping up the use of digital technologies, notably telemedicine (24–26). A 17-country study reports increased use of digital health in mental health care settings, as well as support to facilitate its adoption during the pandemic (27).

Mobile health uses mobile devices such as smartphones to deliver health care. These devices are compact, wireless and universally available at an affordable cost. They provide connectivity, Internet access and multimedia resources at any time and almost anywhere.

The most common mHealth strategies take the form of applications. It is now quite easy to create new applications, find them on online platforms, download them and share them. Mental health applications can be used for Ecological Momentary Assessment (EMA) or Ecological Momentary Intervention (EMI).

EMA is an assessment system that collects data from participants, in their environment and at different times. It includes active data, which generally refers to symptom monitoring questionnaires to be filled in by the individual on their smartphone. It also includes passive data, obtained automatically through sensors on the smartphone or on a wearable device (bracelet or connected watch).

EMI has a similar structure, but the content includes reminders, feedback messages or instructions to adopt specific behaviors or those important for psychotherapy (28). This type of intervention aims to provide support in daily life by sending electronic notifications that encourage therapeutic behaviors at the time they are needed (8). These mobile application-based interventions provide on-demand access to specific therapeutic or psycho-educational tools. Heron and Smyth (29) define these interventions as treatments that are provided to people during their everyday lives (i.e., in real time) and in natural settings (i.e., in the real world). This tool also provides access to a social network. Promising pilot data has been reported for the Moderated Online Social Therapy (MOST) model, an online intervention platform that offers personalized therapy combined with social links and other features. This system combines therapeutic tools, psycho-educational content and a secured, moderated social network. It was developed in Australia by eOrygen, the digital arm of Orygen, The National Center of Excellence in Youth Mental Health, and is led by Professor Mario Alvarez-Jimenez, Chief of Orygen Digital. It is a moderated online social therapy platform that targets 15 to 25-year-olds to improve the social functioning of young people at risk of psychosis (30). The platform can be accessed on mobile phones, tablets and computers. MOST was piloted in Victoria and expanded rapidly in Australia during the COVID-19 pandemic. It consists of a network of peers and expert mental health clinicians. The young patients can also use the platform to interact with other patients.

These tools are used in various ways, ranging from symptom monitoring, medication compliance and promotion of self-management strategies through to access to psycho-education and social relationships.

### 1.4. Current literature and purpose of the review

The evidence to date suggests that smartphone applications could provide an accessible, flexible and inexpensive means of delivering effective self-management interventions for depression and anxiety symptoms (31, 32). A meta-analysis of 18 randomized controlled trials covering 22 mobile applications has shown that using applications for symptom relief significantly reduces patients' depressive symptoms compared with the control group, mainly for people with mild to moderate depression (31). A second meta-analysis of 66 randomized controlled trials found results in favor of intervention groups for depressive symptoms, generalized anxiety and social anxiety (33).

The current published literature on the use of applications in psychosis is more limited. Reviews have highlighted the growing potential of technologies for the treatment of psychosis (26, 34–36) and studies of smartphone applications have shown that they are acceptable and feasible for this population. One review reports the feasibility of using smartphones to improve care for people with schizophrenia, with high rates of engagement and satisfaction over a wide range of applications (34). The FOCUS smartphone intervention study (37) trialed in 2014 on a population of schizophrenic patients shows that the intervention significantly reduces psychotic symptoms.

There are two recent literature reviews on early psychosis: Mar Rus-Calafell and her team summarize the main results of studies between 2009 and 2019 on the use of digital technologies (virtual reality, smartphones and online interventions) to improve the treatment of early psychosis. Most of the studies included are only at the protocol stage and the participants are in an at-risk mental state for psychosis or have FEP (38). The systematic review by Erica Camacho and her team also includes participants in the prodromal and FEP phases. It includes 21 studies: seven papers on protocols, six on feasibility studies, five on validity studies and three on interventions (39). The published literature demonstrates the acceptability and feasibility of interventions. The results show that it is possible to use digital technologies to deliver psychological interventions in the early stages of psychosis, with participants expressing high levels of acceptability and willingness to use them to support their progress and recovery.

To our knowledge, assessment of the therapeutic efficacy of mobile health methods in the management of patients with FEP has not previously been summarized in a systematic review. We have therefore conducted a systematic review of the literature on the preliminary clinical outcomes of digital applications for the support or delivery of treatment for new-onset psychotic disorders. In this literature review, we focus only on patients with FEP and only on one type of technology, mobile smartphone applications that have been developed and validated for acceptability and feasibility. We have focused on studies that assess the preliminary efficacy of this type of tool. In addition, several studies published between 2019 and 2022 have been added. Most of these studies assess the value of adding mobile digital interventions alongside early intervention care or to extend its benefit.

## 2. Method

### 2.1. Eligibility criteria

Following the PICO model, the inclusion criteria were: (i) population representing young patients with first-episode psychosis, no more than 5 years after diagnosis; (ii) intervention using a mobile smartphone application; (iii) in comparison with usual care; (iv) paper written in English or French; (v) describing the effects of mobile health interventions in the management of young patients with FEP and (vi) studies assessing clinical outcomes that could be related to intervention objectives.

The exclusion criteria were: (i) any intervention using technology not provided by a smartphone; (ii) papers not published in English or French and literature reviews; (iii) papers describing experimental protocols with no current results or exploratory studies. Papers including patients in an at-risk mental state for psychosis were excluded, to make the patient sample as comparable and homogeneous as possible.

We have studied smartphone applications rather than other digital platforms because of their mobility and accessibility. We have included studies without a control group due to the fact that this literature is in its infancy.

### 2.2. Literature search strategy

A systematic search of the international literature was carried out using the Pubmed and Embase search engines. The search covered papers published between the creation of the database and 13 May 2022 using a search equation including the following Medical Subject Headings (MESH): (Psychosis OR Schizophrenia) AND (Early Medical intervention) AND (Digital Technology OR smartphone OR mobile applications OR social media OR internet). The references cited in the selected papers were reviewed to identify any additional relevant studies.

### 2.3. Study selection process

**Figure 1:**
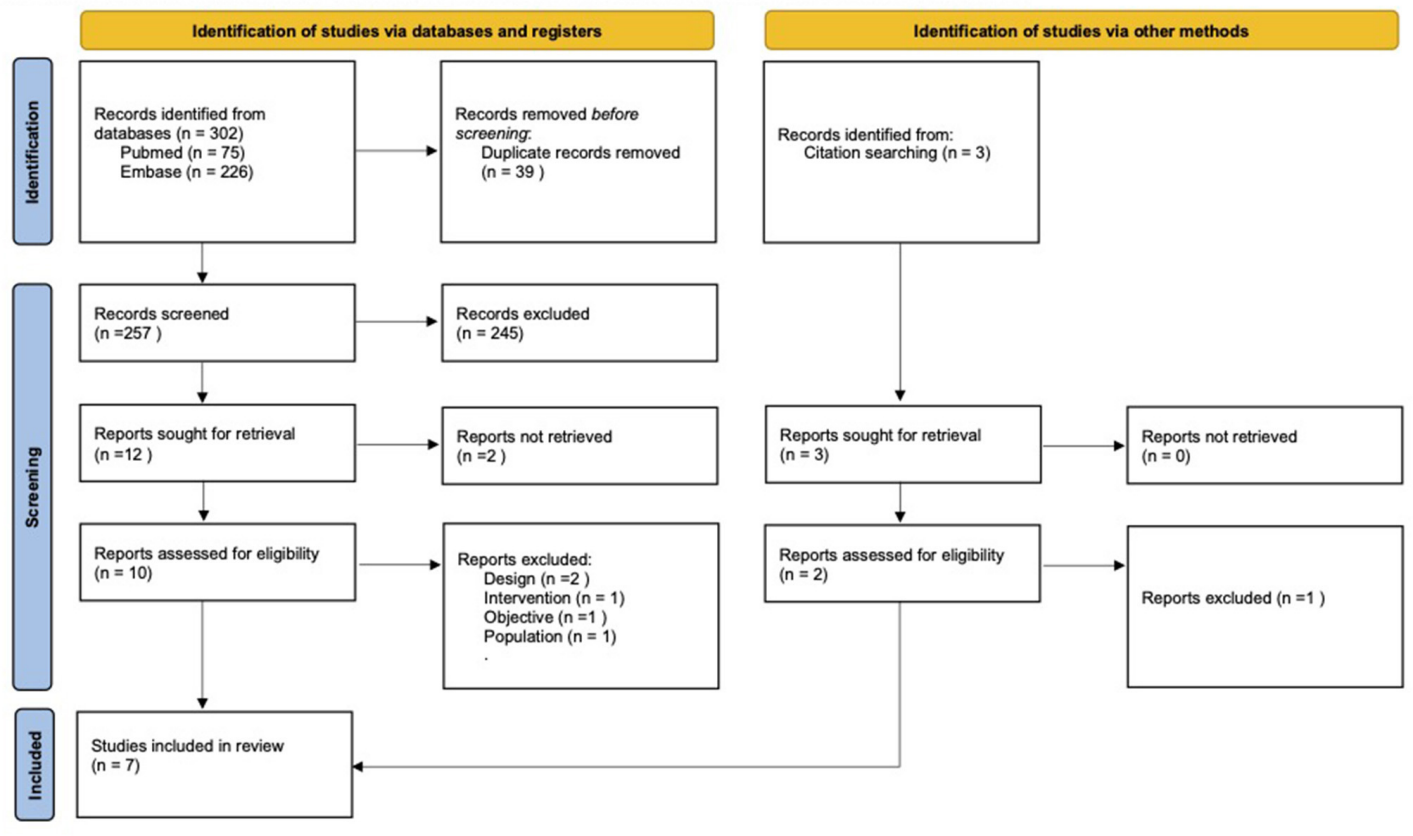
Flow chart of study selection according to PRISMA 2020 guidelines.

Two authors (CM and AC) screened the titles of the publications identified in the databases using the search strategy defined above to identify potentially eligible studies. Both authors, first independently and then jointly, screened the studies based on their abstracts. All online abstracts were reviewed and full-text papers were retrieved where relevant. In case of disagreement, a third author (AY) was called on to arbitrate. This search procedure followed PRISMA 2020 (Preferred Reporting Items for Systematic Reviews and Meta-Analyses) guidelines (40) (Figure 1).

### 2.4. Bias assessment

To our knowledge, no rigorously validated method of assessing the quality of studies in this field exists. We applied the method using the Jaddad scale (41) that includes: randomization, masking/double blind and a description of losses during follow-up. The variable of the existence or not of a “control group” was added as done by Bonet et al. in the 2017 review to conduct a reproducible analysis of the study quality (18).

The assessment is based on the presence or absence of the following criteria:

1. Randomized.
2. Double blind.
3. Losses.
4. Sufficiently randomized (studies that indicate the randomization technique used (computer-generated table of random numbers, throwing a coin, properly shuffled envelopes, etc.).
5. Sufficiently double blind.
6. Control group.
7. Total.

We considered studies to be of poor quality when they scored < 3 points, and they were considered to be of maximum quality at 5 and 6 points.

## 3. Results

### 3.1. Selection of studies

The flow chart (Figure 1) describes the study selection process and shows the initial selection of 302 papers and then the selection of 257 papers for in-depth assessment after eliminating duplicates. Two hundred forty-five further papers were excluded after review of their titles and abstracts. Full-text versions were retrieved for 12 papers, of which five were eligible for inclusion. Two further papers were added from the references cited in the papers studied. Hence, a total of seven studies have been included in this review.

### 3.2. Characteristics of the selected studies

The full details of each study are shown in Table 1 (and Supplementary Data S1). Results were available for four randomized controlled trials, one pragmatic clinical trial and two open trials. The average age of the sample was 24.4 years. Smartphone interventions lasted for an average of 25 weeks. Table 1 summarizes the papers included and their characteristics.

**Table 1:** Characteristics of seven studies on mobile health (mHealth) in the treatment of first-episode psychosis.

**References**	**Application**	***N***	**Mean age**	**Population origin**	**Design**	**Objective**	**Intervention**	**Results**
Lewis et al. (42)	ClinTouch	44	26.1	“Early psychosis” sub-group: patients receiving care in an EIP programme In the first three years following FEP London (United Kingdom)	Two-center open-label randomized controlled trial Active monitoring of symptoms under ClinTouch plus treatment as usual compared with treatment as usual alone Length of intervention: 12 weeks	Assess the acceptability and safety of continuous monitoring for 3 months, the impact on positive psychotic symptoms at 6 and 12 weeks, and the feasibility of detecting early signs of relapse by the care team through the application	EMA Active symptom monitoring Alerts sent to the care coordinator when personalized warning-sign thresholds were exceeded	Significant reduction in positive PANSS in the “early intervention center” sub-group (adjusted mean difference −3.04; CI −5.49, −0.59; *p* = 0.016) The performance of the prototype early warning signs algorithm was “sub-optimal” with a sensitivity of 75%, a specificity of 8%, giving a positive predictive value of 29%
Bonet et al. (43)	ReMind Care	90	32.8	17–65 years old Patients receiving care in an EIP programme since 2018 Within 5 years after FEP Valencia (Spain)	Pragmatic clinical trial comparing ReMindCare with TAU over 19 months Length of intervention: 50 weeks	Assess the efficacy and clinical results of application use in terms of adherence to ReMindCare, prevention of relapse, hospital admissions and A&E visits	EMA ReMindCare offers active symptom monitoring and alerts to doctors in case of low engagement or sudden changes to questionnaire replies	Significantly fewer relapses (χ^2^ = 13.7, *p* = 0.001), hospitalizations (χ^2^ = 4.6, *p* = 0.03) and A&E consultations (χ^2^ = 7.4, *p* = 0.006) in the ReMindCare group
Alvarez-Jimenez et al. (44)	Horyzons	170	20.9	16–27 years old. After 18–24 months' care in an Early Psychosis Prevention and Intervention Center (EPPIC) Melbourne (Australia)	Phase 4 randomized controlled trial, parallel groups, single blind Compare Horyzons plus TAU with TAU alone Length of intervention: 18 months (72 weeks)	Assess whether digital intervention is an effective strategy to extend the benefits of EIP treatment and promote social and vocational recovery beyond discharge from these specialized EIP programmes and prevent relapse after FEP	EMI Horyzons is based on the MOST model	No significant change in social functioning (PSP score; mean difference = −0.29, 95% CI: −4.20 to 3.63, effect size = −0.01, *p* = 0.77) or in secondary endpoints (CDSS, UCLA, MOS-SSS, SERS-SF, MHCS, SWLS, AQoL, PANSS). 5.5 times more likely to find a job and/or enroll in education (OR = 5.55, 95% CI: 1.09–28.23, *p* = 0.04). Significantly less likely to visit A&E (p = 0.03) twice as many hospitalizations for psychosis in the TAU group, without significant difference
Ludwig et al. (12)	Horyzons	26	24.9	18 to 35 years old, cared for by three EIP departments in North Carolina (United States)	Uncontrolled open trial Length of intervention: 12 weeks	Assess acceptability and feasibility, assess whether participation in this platform correlates with a reduction in the feeling of loneliness, and an improvement in social integration and the feeling of wellbeing	EMI Horyzons is based on the MOST model	Improvement in PANSS psychosis-related symptoms (*d* = 0.81). Moderate reduction in UCLA experience of loneliness (*d* = 0.27), BDI depressive symptoms (*d* = 0.30), and mDES NEG negative emotions (*d* = 0.27). Login frequency was significantly associated with improved psychological wellbeing in actively engaged participants
McEnery et al. (45)	Embrace	10	23	Participants in the Horyzons study after 2 years' EIP care and 18 months' online social support (Horyzons)	Single-arm open-label trial Length of intervention: 8 weeks.	Assess the feasibility, acceptability and safety of online intervention using Embrace, designed to treat social anxiety as the primary target in young people with FEP	EMI Embrace is based on the MOST model The clinical content targets a specific CBT therapeutic goal related to management of social phobia	Statistically significant decreases for social anxiety symptoms with SIAS (*d* = −1.70, *p* = 0.0005) and LSAS (*d* = −1.35, *p* = 0.002). Non-statistically significant decreases for depression (*d* = −0.22, *p* = 0.50) and loneliness (*d* = −0.23, *p* = 0.48; DASS and UCLA)
Schlosser et al. (16)	Prime	43	24	16–36 years old with new-onset schizophrenia spectrum disorder, first 5 years of illness (United States)	Randomized controlled trial comparing the Prime group with the TAU control group Length of intervention: 12 weeks	Assess the ability of the application to improve motivational disorders during first-episode psychosis	EMI Designed to target motivation by setting goals to be achieved, with individualized CBT-based follow-up and coaching The application's interventions consisted of automated reminders of goals and challenges and real-time messaging with a clinician	Significant improvements in favor of the intervention group were found for two motivational components (anticipated pleasure and effort expenditure). Respectively: *F*_(1, 56)_ = 4.75, *p* = 0.03 and *F*_(1, 56)_ = 4.66, *p* = 0.04 A trend toward significant improvement in reward learning was found *F*_(1, 56)_ = 3.53, *p* = 0.07 Significant differences for defeatist beliefs, *F*_(1, 57)_ = 5.58, *p* = 0.02, for depressive symptoms, *F*_(1, 56)_ = 7.06, *p* = 0.01, and for feelings of self-efficacy, *F*_(1, 55)_ = 5.76, *p* = 0.02 No differences in changes in positive or negative symptoms (PANSS), quality of life (QOL-A) or functioning (RFS)
Bucci et al. (22)	Actissist	36	19.3	>16 years old Within the first 3 years after FEP Cared for in an EIP programme North-west England	Pilot single-blind randomized controlled trial comparing Actissist plus TAU with ClinTouch plus TAU Length of intervention: 12 weeks	Assess the safety, feasibility and acceptability of Actissist intervention Provide preliminary evidence of the effects of the intervention on clinical and functional outcomes	EMI compared with EMA (Active control) Intervention based on CBT tools	Greater improvement in negative symptoms (negative PANSS), general psychotic symptoms (general and total PANSS) and mood (Calgary score) in the Actissist + TAU group compared with the ClinTouch + TAU group with numerically higher regression coefficients (adjusted mean differences) and standardized effect sizes (Cohen's *d*) for the post-treatment assessment with Actissist

EIP, Early Intervention in Psychosis; FEP, first-episode psychosis; EMA, Ecological Momentary Assessment; EMI, Ecological Momentary Intervention; MOST, Moderated Online Social Therapy; TAU, treatment as usual; PSP, Personal and Social Performance Scale; BDI, Beck Depression Inventory; mDES Neg, modified Differential Emotions Scale - Negative Sub-scales; AQoL, Assessment of Quality of Life; CDSS, Calgary Depression Scale for Schizophrenia; UCLA, UCLA Loneliness Scale; MOS-SSS, Medical Outcomes Study Social Support Survey; SERS-SF, Self-Esteem Rating Scale - Short Form; MHCS, Mental Health Confidence Scale; SWLS, Satisfaction with Life Scale; AQoL-8D, Assessment of Quality of Life - 8D; PANSS, Positive and Negative Syndrome Scale; RFS, Role Functioning Scale; SIAS, Social Interaction Anxiety Scale; LSAS, Liebowitz Social Anxiety Scale.

The seven studies assessed smartphone interventions focusing on symptoms monitoring, therapeutic intervention and/or social networks.

### 3.3. Quality

No study attained the highest score for methodology, as the lack of blind was their main limitation. In addition, four studies scored < 3 and were considered to be of poor quality (Table 2). Only four of the studies were randomized controlled trials (16, 42, 44, 46), one out of four was from a sub-group (47). The samples included in this study are small and the follow-up periods short. One study used a sample from a previous intervention.

**Table 2:** The methodological quality of studies analyzed.

**References**	**Application**	**Randomized**	**Double blind**	**Losses**	**Sufficiently randomized**	**Sufficiently double blind**	**Control group**	**Total**
Lewis et al. (42)	ClinTouch	1	0	0	1	0	1	3
Bonet et al. (43)	ReMind Care	0	0	0	0	0	1	1
Alvarez-Jimenez et al. (44)	Horyzons	1	0	1	1	0	1	4
Ludwig et al. (12)	Horyzons	0	0	1	0	0	0	1
McEnery et al. (45)	Embrace	0	0	0	0	0	0	0
Schlosser et al. (16)	Prime	1	0	0	0	0	1	0
Bucci et al. (22)	Actissist	1	0	1	1	0	1	4

0 = No; 1 = Yes.

### 3.4. Symptom monitoring and improvement

The symptom monitoring studied here consists of daily assessment of patients' state of health through short questionnaires covering positive psychotic symptoms, anxiety and mood. The mobile applications can monitor symptoms and may include a secure portal where the clinician receives clinical information. The clinician is thus aware of changes in symptoms and can adjust interventions accordingly, in addition to conventional clinical follow-up (48). Lewis et al. did not show any significant difference between the groups in the total positive PANSS after 6 and 12 weeks, and no difference in secondary endpoints. However, after a separate intention-to-treat analysis for each site, the study shows a significant reduction in positive PANSS after 12 weeks of ClinTouch monitoring in the Early Intervention sub-group (adjusted mean difference −3.04; CI −5.49, −0.59; *p* = 0.016). The results regarding the performance of the prototype early warning signs algorithm are “sub-optimal” for the accuracy of ClinTouch alerts compared with the warning signs as documented in the electronic patient record, with a sensitivity of 75%, a specificity of 8%, giving a positive predictive value of 29% (42). Bonet et al. (43), showed that after 19 months of using ReMindCare, only 20% of patients in the ReMindCare group suffered a relapse, while 58% of TAU patients had one or more relapses (χ^2^ = 13.7, *p* = 0.001). In addition, ReMindCare patients had fewer urgent care unit visits (χ^2^ = 7.4, *p* = 0.006) and fewer hospitalizations than TAU patients (χ^2^ = 4.6, *p* = 0.03). Of the 59 ReMindCare patients, 31% requested an urgent consultation, 20% relapsed while using the application and 8% developed a delusion involving the application and the research group. After 19 months of intervention, 63% of patients continued using the application, while 12% stopped using the application because they were discharged from the EIP department and 25% opted to stop using ReMindCare. Reasons for discontinuation: 33% of patients felt suspicious about the technology (among these patients, 4 had a relapse while using the application); 40% (6/15) perceived the application as boring and did not perceive any benefit; and 27% (4/15) of patients left treatment and did not continue in the programme. The 2018 Actissist (46) showed greater improvement in negative symptoms (negative PANSS), general psychotic symptoms (general and total PANSS) and mood (Calgary score) in the Actissist + TAU group compared with the ClinTouch + TAU group with numerically higher regression coefficients (adjusted mean differences) and standardized effect sizes (Cohen's *d*) for the post-treatment assessment with Actissist. The effects were not fully maintained at the 22-week follow-up, though there was no decline in any of the clinical outcomes measured. Focusing on Horyzons project, there was no significant difference in the hospitalization rate and no significant difference in psychotic symptoms. There were no significant changes in secondary endpoints (psychotic symptoms measured by PANSS, depressive symptoms measured by the Calgary Depression Scale for Schizophrenia CDSS, selfesteem measured by the Self-Esteem Rating Scale SERS-SF, selfefficacy measured by the Mental Health Confidence Scale MHCS). In addition, participants assigned to Horyzons were significantly less likely to visit A&E over the 18-month period (*p* = 0.03) compared with the TAU group. The TAU group had twice as many A&E visits as the Horyzons plus TAU group from baseline to 18 months, a statistically significant difference (39 vs. 19% respectively; OR=0.31, 95% CI: 0.11–0.86, *p* = 0.03, NNT = 5).The TAU participants had twice as many hospitalizations for psychosis as the Horyzons plus TAU group, without significant difference (27 vs. 13% respectively; OR = 0.36, 95% CI: 0.11–1.08, *p* = 0.07, NNT = 7). In Horyzons US (12), the results showed an improvement in psychosis-related symptoms (PANSS): large effect size from baseline to mid-treatment (Cohen's *d* = 0.81) and medium to large effect size from baseline to end of treatment (Cohen's *d* = 0.65). They also showed a moderate reduction in experiences of depressive symptoms and negative emotions after 6 weeks of using the platform. Self-reported experience of negative emotions (mDES NEG): small to medium effect size between baseline and end of treatment (Cohen's *d* = 0.27). Depressive symptoms (BDI): small to medium effect size between baseline and mid-treatment (Cohen's *d* = 0.30). McEnery et al. (47) using Embrace, showed a statistically significant reduction in social anxiety symptoms as measured by the Social Interaction Anxiety Scale [SIAS, (49)] between baseline and the end of the intervention (*d* = −1.70, *p* = 0.0005). This significant reduction is also confirmed using the Liebowitz Social Anxiety Scale (50), (*d* = −1.35, *p* = 0.002). Finally, non-statistically significant decreases were found for depression (*d* = −0.22, *p* = 0.50) the secondary endpoint. Participants reported that the application provides them with a sense of control over their social anxiety symptoms.

The 2018 Prime group (16) did not highlight differences between the groups in changes in positive or negative symptoms (PANSS). In addition, there was a trend toward significant improvement in reward learning, *F*_(1, 56)_ = 3.53, *p* = 0.07 and the results showed significant differences for defeatist beliefs, *F*_(1, 57)_ = 5.58, *p* = 0.02, for depressive symptoms, *F*_(1, 56)_ = 7.06, *p* = 0.01.

Three interventions can monitor symptoms (42, 43, 46). Except Horyzons that did not show any difference focusing on symptoms (44), the others interventions were associated with improvement of general (12, 46), positive (42, 46), negative symptoms (46), mood (12, 16, 46) and anxiety (47). One intervention was associated with a reduction of hospitalization (43) and A&E visits (43, 44).

### 3.5. Social network and interaction

Horyzons (44) incorporating a moderated social network aimed at recovery after FEP is, to date, the most advanced online psychosocial intervention programme for early psychosis. However, the results showed no difference in social functioning, the primary endpoint. There was no significant change in Personal and Social Performance Scale (PSP) scores at 18 months follow-up (mean difference = −0.29, 95% CI: −4.20 to 3.63, standardized effect size = −0.01, *p* = 0.77). The level of functioning remained stable for both groups between the start and 18-month follow-up. However, patients in the Horyzons intervention group were 5.5 times more likely to find a job and/or enroll in education compared with the TAU group (OR = 5.55, 95% CI: 1.09–28.23, *p* = 0.04).

According to a *post-hoc* analysis, participants in the top quartile of logins (i.e., logging in >77 times) show greater improvement in employment and education outcomes (OR = 59.71; 95% CI: 2.40–1484.37, *p* = 0.01) compared with those in the bottom quartile of logins (i.e., < 9 logins; OR = 1.40; 95% CI: 0.03–72.40, *p* = 0.87).

In US version (12), focusing on loneliness (UCLA), they highlighted small to medium effect size between baseline and mid-treatment (Cohen's *d* = 0.27). Login frequency is significantly associated with improved psychological wellbeing in actively engaged participants. Minimum use of the platform is defined as an average of at least one login per week (12 logins in total) and at least 10 uses of the application (e.g., comments, talking points, etc.). The rate of active participants (patients who met or exceeded this threshold) is 79%, while the rate of inactive participants (patients who did not meet the minimum usage) is 21%. The *Embrace* programme (45) was associated with a non-statistically significant decreases of loneliness (*d* = −0.23, *p* = 0.48) scores.

None study showed an impact on functioning or loneliness scale. However, Horyzons program was associated with a return to working life.

### 3.6. Engagement

Most of the programs were associated with an engagement range from moderate to high. Six studies highlighted an engagement over 70% (16, 42–44, 46, 47). Moreover, Preliminary findings suggested active engagement in Horyzons was associated with enhanced social integration, improved psychological wellbeing, increased positive emotions, as well as decreased negative emotions and depressive symptoms (12). However, the methods for measuring engagement with the applications were different in each study, making comparisons difficult.

## 4. Discussion

The results of this systematic review provide preliminary evidence for the efficacy of digital mobile applications in the treatment of young patients with FEP. Our review highlighted that mobile health can be used to monitor symptoms (42, 43, 46). Most of programs were associated with an improvement of symptoms (12, 16, 42, 43, 46, 47) and an acceptable engagement (16, 42–44, 46, 47). MHealth seemed have an impact on hospitalization (43) and A&E visits (43, 44) on the one hand and on the return to working life, on the other hand (44). In addition, none program was associated with functioning or social scale.

Regarding interventions based solely on EMA (symptom monitoring) methods, one study found that remote monitoring of symptoms minimized relapses, A&E visits and hospitalizations. The second indicated a reduction in positive psychotic symptoms.

Concerning EMI interventions, one study found an improvement in anxiety symptoms and two studies noted an improvement in psychotic symptoms. One EMI demonstrated its efficacy in helping participants return to studying and employment. One EMI study reported improved motivation. Two out of four studies found no significant changes in psychotic symptoms.

The studies have demonstrated the promising potential of applications in the recovery phase after FEP. They could facilitate self-management of the illness through symptom monitoring by providing instructions for self-management of symptoms in daily life. The addition of therapy modules increases access to evidence-based tools, improves the quality of treatment, facilitates goal achievement and encourages autonomy.

Nevertheless, there are several limitations. Chief among them are internal validity and power.

Moreover, these results should be interpreted with caution as two trials were not controlled, one trial was not randomized (PCT) and we included a sub-group from one trial in an intention-to-treat analysis.

Four studies scored < 3 and were considered to be of poor quality and the three others were considered as moderate quality. These methodological limitations reflect the fact that this field of research is in its infancy. A number of studies identified as relevant for this review were excluded as they did not present preliminary clinical outcome data. They highlight the feasibility and acceptability of a range of additional applications that use the EMA and EMI methods.

It should be noted that the methods for measuring engagement with the applications were different in each study, making comparisons difficult. In addition, engagement may have been encouraged (e.g., through a financial incentive) which may have increased the take-up rate.

Barriers to the implementation of digital technologies in mental health are highlighted (51–54), particularly in the care of new onset psychosis. A number of practical issues are described in relation to digital interventions in psychiatry, including the cost of installing and maintaining equipment and software (technical support, storage, data analysis and technology upgrades), the ability of healthcare IT infrastructures to adapt to new technologies and incorporate them into clinical practice. Cost-effectiveness data has not yet been reported. In addition, new technologies are not progressing at the same pace as clinical trials, which may affect the acceptability of digital interventions (52). A review of engagement with popular commercially available mental health applications found that only 4% of users who downloaded a mental health application reopened it after 15 days (55).

In a paper that reviews the challenges surrounding user engagement with smartphone mental health applications (56), the authors state that “low engagement,” in other words poor adherence to the intervention, represents a major barrier to widespread use of these technologies. In addition, there is no uniformity in the measurement of engagement across the studies. There are no standard measurements to compare engagement with applications in the various papers published and often engagement data is not reported.

Moreover, digital technology tools also raise important ethical issues regarding informed consent, confidentiality, data protection and patient privacy. These factors are all the more important when vulnerable populations and private health information are involved. This is problematic when digital health tools involve the collection of massive amounts of personal data. In a study of the views of patients with psychotic disorders on mobile health, Their first concern was privacy (followed by the reliability of the application) (57). Another study suggests that people with mental health problems are less comfortable with automatically sharing personal data (58). This suggests the importance of a patient-centered approach, and of working closely with future users from the start of any project. A 2016 review examined the specific features of 208 mental health applications and found that only 9% provided data security or privacy protection and 89% made no mention of it. Fifty-nine percent of the applications provided no information on the efficacy of the application (59).

In addition, in 2017, more than 10,000 mental health-related applications were commercially available (60), yet despite the proliferation of health applications, few can be considered to be of good quality. There is currently limited evidence on the value and robustness of the theoretical foundations provided by the applications (61). Research in 2019 found that only 3% of commercially available mental health applications had an evidence base to support their claims of efficacy (61, 62). In addition, access to these interventions is still restricted for non-English speaking populations.

There is no regulation or consensus on health applications and few guidelines or standards on which to base application research and quality assessment. The disparity in the quality of mobile applications has stimulated the development of assessment tools. The American Psychiatric Association (APA) has developed a model to help clinicians improve informed decision-making about mental health applications: the “APA App Advisor” (63). It assesses the accessibility, confidentiality, safety, clinical basis, ease of use and data integration of the tool in terms of a therapeutic goal.

The *Mobile Application Rating Scale* (MARS) is another tool that aims to provide a standardized assessment of mental health applications for clinicians (64). The “uMARS” version has been developed for users. In 2019, the World Health Organization (WHO) published a guide to pilot and assess digital solutions and help harmonize practices, with summary tables of the different methodological approaches according to the clinical assessment goals of these applications.

Nevertheless, it remains difficult for users and clinicians to identify the quality and usefulness of the applications available.

In future, passive data and personalized interventions could improve the use of mHealth. The term “active data” refers to data generated through the active involvement of a patient, such as self-questionnaires, while “passive data” refers to data generated without the patient's involvement (GPS, accelerometer, voice calls and SMS) (65). The many sensors built into smartphones offer a wealth of data, such as GPS to monitor spatial location, an accelerometer to record movement and overall motor activity, call and messaging histories to document social activity, voice and sound recordings to estimate mood, a camera for facial expression. The sensors may be on the smartphone or on a wearable device (a connected watch or bracelet). Often referred to as “digital phenotyping,” passive monitoring provides a means of understanding mental health experiences in context.

Qualitative data from studies on the ClinTouch application report that some patients find the self-questionnaires repetitive (66). This may lead to disengagement in the longer term. Researchers are currently investigating whether passive monitoring of psychotic relapse indicators using sensor technologies embedded in smartphones may be more acceptable to users and more responsive to change than active self-assessment. The Crosscheck system (67) is currently being developed and tested by Dr Ben-Zeev's team, to detect changes in speech properties, physical activity and location to generate personalized alert patterns. An early prototype of the system appears to be acceptable to participants with psychosis, although the research team notes that self-selection is likely: those who are concerned about such monitoring will choose not to participate in the tests. If successful, Crosscheck could be used to signal a potential relapse and trigger an early intervention response in the same way as ClinTouch (52). The Crosscheck application combines the use of active EMA data with passive data such as physical activity (accelerometer), geospatial activity (GPS), speech frequency and duration (microphone) and phone use (telecommunication, application use, screen unlocking) to predict relapse in people with psychosis. The results of the study reveal that the digital indicators of relapse are not the same for each person with psychosis.

In the study by Cella et al. (68), the investigators combine active and passive digital technology using a wrist-worn device (the Empatica E4) and the ClinTouch application. The study assessed whether there was a link between psychotic symptoms and a physiological response. The results showed increased electrodermal activity during hallucinations or delusions, but no association between symptoms and heart rate variability. This study suggests that it may be possible to identify a reliable biosignature indicating worsening symptoms and a risk of relapse.

Other research supports the feasibility of digital phenotyping in psychotic disorders, such as the study of the Beiwe application (69). This study suggests that 2 weeks before relapse, people with schizophrenia show significant changes in mobility indicators derived from GPS data, sociability indicators derived from text messages and call data, and symptom exacerbation indicators derived from self-assessment surveys within the application. This indicates that it may be possible to capture digital indicators of relapse. The rate of behavioral abnormalities detected in the 2 weeks prior to relapse is 71% higher than the rate of abnormalities in other periods.

As Torous points out (51), this could help to understand the heterogeneity of clinical presentation and offer a more personalized understanding of psychotic illness. Smartphones and other portable devices can now capture real-time environmental data on behavioral indicators. This data offers potential insights into how symptoms can lead to clinical presentations such as social withdrawal and anhedonia via changes in call/text reciprocity, or avolition and lethargy via changes in GPS-tracked movements (70). This wealth of readily available information offers a new perspective to better characterize the lived experience of people with FEP and to explore new subtypes and clusters of psychoses based on new data.

Digital technologies could also be used to predict illness trajectory: smartphones and related mobile devices offer a means of capturing daily fluctuations in the multitude of indicators needed to better understand, model and predict the trajectory of the illness. It remains to be seen whether this means of data capture is acceptable to users and whether it risks increasing the symptoms of paranoia in people with psychotic disorders.

This objective measure of digital phenotyping occurs in the context of patients' lived experience, reflecting how they function in their environment. The smartphone may be an opportunity to measure real-world functioning and potentially offer real-time interventions (71). In just-in-time adaptive intervention (JITAI), active and passive data is collected to help develop personalized, real-time intervention strategies. For example, the smartphone can deduce low mood in the context of social isolation and suggest a relevant intervention, while if it deduces low mood in the context of poor sleep, it can recommend an alternative intervention. While still in its infancy, the use of JITAI to deliver mental health interventions would be an interesting area for future research (72).

A major opportunity offered by digital technology is to improve engagement, especially among young people. Gamification is promising in this respect (73), e.g., by adapting the techniques of digital gaming, offering rewards and challenges to complete activities. The most commonly observed gamification features in mental health are: progress tracking; points; rewards; introduction of themes or stories; personalization; configuration (74). The addition of gaming features could support engagement, increasing motivation, creating a sense of empowerment and inducing positive emotional responses in users, such as a sense of pride.

The use of mobile applications for smartphones represents an interesting prospect for improving the engagement of FEP patients receiving care (75). A meta-analysis (76) assessing the opinions of 1,172 psychotic patients on mHealth services reports that 60.2% of users are in favor of using mobile phones to track and monitor their mental health and 51.1% to facilitate contact with health professionals. The study shows that this population is interested in this type of tool to facilitate the link between patient and healthcare department.

In a study assessing interest in new technologies among psychotic patients, the service of most interest to patients was the “contact alarm to clinicians in case of emergency” (77). Patients demand more personalized, more interactive and closer clinical attention. However, as noted in the studies, when it comes to the clinical implications associated with these interventions, it is very important to design these systems from the clinician's perspective. In a study exploring the attitude of mental healthcare staff in England to digital health interventions, staff expressed concern about their moral, legal and professional obligations in relation to assessing information about risks such as suicidal ideas and behavior. They preferred patients to report symptoms themselves during consultations. Not only did staff feel that this would give patients control over the information they share, but also that their level of responsibility would be minimized. This somewhat contradicts the current focus of smartphone applications for symptom monitoring in this population, which, although they can be used by patients to share with their care team, tend to provide symptom reports to a central server that staff can use to identify early signs of relapse. Issues surrounding the legal and moral responsibilities of staff when viewing automatic symptom reports and their level of comfort in implementing such approaches in practice must be considered (78).

These various limitations need to be considered when jointly building new technologies with the people affected by FEP to ensure that the technologies are appropriate and that they will be used. Among the possible solutions identified to increase young people's interest in quality applications, the inclusion of users in the development process is essential, involving them in development of the objectives, the planned functions and the design of the application.

One prospect that shows promise concerns case management follow-up with a mobile application used jointly by patients and case managers. The Heal Your Mind application (79), currently under development, offers case management based on CBT techniques and symptom monitoring for young people with FEP. Surveys have shown that most participants use at least five of the six modules, find the application easy to use and express satisfaction with the tool. The feature that is most frequently used, most highly appreciated and perceived as most useful is communication with the case manager.

The French Plan-e-Psy project, led by Dr Frederic Haesebaert, aims to work with people affected by FEP and their families to jointly build a monitoring application in the context of *case management*. The protocol describes improvement in patient functioning as the primary endpoint (80). It aims to allow both the case manager and the patient to plan and monitor the achievement of individualized care goals. The assumption is that the use of such an application will improve the functioning of patients receiving care for FEP. This randomized, multi-center clinical trial will include 168 participants aged 18–30 with first-episode psychosis. The results are expected in January 2024.

## 5. Conclusion

Schizophrenia is one of the most severe psychiatric illnesses. It is frequent and still too often incapacitating. Clinical research in recent years has shown that early intervention leads to a more favorable evolution of this illness. Mobile health could also have a role to play in changing and enhancing the quality of care for psychosis. It could provide new opportunities to increase access to existing mental health resources, improve the quality of treatment and enhance the provision of mental health care. The future of mobile technology in mental health care is indeed gaining momentum in the literature. Preliminary data on efficacy is emerging in the literature.

Mobile health could help make users more autonomous and take greater responsibility for their own care, through monitoring and assessing symptoms and facilitating self-management strategies. This autonomy does not imply the replacement of health professionals, but would instead optimize their contribution by providing assistance in caring for patients. It could promote patient involvement in healthcare in general by encouraging them to be actors in their own care, enhancing their empowerment.

The use of technology for therapeutic purposes has the potential to increase access to standard treatments and to allow greater patient choice and control. It can offer the choice of a wide range of therapeutic interventions, personalized resources, psycho-education and various types of specialized therapy tools. These interventional applications can be personalized to address individual issues in real time and promote functional recovery. They could also facilitate peer support and social integration by providing secure social networking platforms.

Applications could be clinically incorporated into existing healthcare environments to provide patients with FEP with new tools and ways to engage in care by facilitating connections to clinical care. Smartphone-based symptom monitoring could be incorporated into electronic patient record systems and regular clinical monitoring to generate clinically usable information and predictions for preventive and personalized care. New models of healthcare could take advantage of these technologies while preserving the therapeutic relationship and including patients in the tool development process.

The literature highlights many of the challenges facing this new field of research, including ethical issues, cost, and the capacity of healthcare infrastructure, along with doubts about the quality of applications on the market. The rapid pace of technological development is at odds with the long scientific process required to develop a quality application. Clinical evidence for the efficacy of applications is currently limited. The lack of quality validation is one of the main problems. The rapid expansion of the mobile health sector makes it difficult for users to choose and for professionals to recommend the right application. On the other hand, these technological advances can make applications more attractive and improve results. To facilitate this, future research can explore barriers and potential solutions, focusing particularly on feedback from users and healthcare providers.

Mobile applications are emerging as interesting tools for better engagement in care, enhanced self-management of symptoms and better coordination of resources. This systematic review has several limitations due to the lack of randomized controlled studies. Overall, the studies to date suggest promising preliminary efficacy data on the use of mobile applications in early psychosis. Given the importance placed on early intervention in psychosis, the implementation of technologies for therapeutic purposes in young adult populations in the early stages of psychosis seems essential. While engagement is a challenge in traditional clinical practice, technological progress can enhance the engagement of young people in particular.

## Data availability statement

The original contributions presented in the study are included in the article/Supplementary material, further inquiries can be directed to the corresponding author.

## Author contributions

CM and AC conceived the study and screened the titles of the publications identified in the databases using the search strategy defined above to identify potentially eligible studies. Both authors, first independently and then jointly, screened the studies based on their abstracts. All online abstracts were reviewed and full-text papers were retrieved where relevant. In case of disagreement, a AY was called on to arbitrate. CM wrote the initial draft and all tabular material. AC and AY supervised the study and critically revised the manuscript. All authors read and agreed to the published version of the manuscript.
