# Supplementary material for: Mobile health in the specific management of first-episode psychosis: a systematic literature review
Source: Front Psychiatry. 2023 Jun 12;14:1137644. doi: 10.3389/fpsyt.2023.1137644 (PMC10291100; doi:10.3389/fpsyt.2023.1137644)
Supplement: Supplementary file 1 [file Data_Sheet_1.docx]

## Characteristics of the selected studies

ClinTouch is a mobile assessment application that uses EMA to assess the symptoms of patients with psychotic disorders by self-monitoring. This mobile application was developed to reduce the occurrence of severe psychotic episodes. The system invites users to answer a number of questions about their symptoms, throughout the day and in their real-life environment. It aims to encourage psychotic patients to take an active role in their care by monitoring their emotional and psychological state in real time. The software enables remote monitoring of symptoms and provides continuously updated information in real time, allowing professionals to monitor patients’ symptoms remotely and receive personalised alerts when symptoms exceed a previously agreed symptom threshold(41). The system aims to manage the data collected by recording it in a database, to monitor symptoms and potentially allow early warning of a relapse to be given to the healthcare team. The result is an individual personalised plan of the risk of relapse. When significant symptoms are detected, the application sends alerts to the medical teams. Depending on the severity of the symptoms, they may decide to intervene quickly by scheduling a medical appointment with the patient. The questionnaire assessment items have been designed to be equivalent to 12 PANSS items and 2 items on the Calgary Depression Scale (CDS). The care coordinators determine the criteria for warning signs of relapse with each participant beforehand to define a critical threshold, and an alert algorithm has been developed. Among symptom monitoring applications for psychotic disorders, the best known is ClinTouch, Lewis et al. (42) compared the use of the ClinTouch smartphone application with “treatment as usual” (TAU). This randomised controlled trial has three objectives: to assess the acceptability and safety of continuous monitoring for 3 months, the impact of self-monitoring on positive psychotic symptoms at 6 and 12 weeks, and the feasibility of the algorithm for detecting early signs of relapse by the care team. Patients were recruited from two different centres, either an early intervention department or a community mental health department.

ReMindCare is an application for daily assessment of the state of health of patients with early psychosis through the use of short self-questionnaires. Daily questions assess levels of anxiety, sadness and irritability and weekly questionnaires assess medication adherence, presence of side effects to antipsychotic medication, attitude towards taking medication and presence of psychotic symptoms. Patients also have access to a chart showing the change in their symptoms. In addition, the application provides predefined alerts in case of low engagement or sudden changes to questionnaire replies. Physicians are notified of the alerts by email and they are also displayed in the patient profile on the application’s website portal. Patients can contact their referral psychiatrist directly in case of worsening symptoms by using the urgent consultation request tab on the application home screen. If they click on the urgent consultation request, their doctor will contact them by telephone within 48 hours. In addition, clinicians communicate with patients by telephone in response to the predefined alerts. Urgent care consultations can be scheduled if necessary (43). Bonet et al., in a 19-month pragmatic trial (in the real world), compared the ReMindCare application with treatment as usual in Spain. It assesses efficacy and clinical outcomes in terms of adherence to the application, relapse prevention, hospital admissions and Accident and Emergency (A&E) visits compared with treatment as usual without the application. Participants ranged in age from 17 to 65 (mean age 32.8 years), had a diagnosed psychotic disorder with less than 5 years of illness and were part of an EIP programme. (43).

Horyzons is an online programme that incorporating a moderated social network and psycho-education aimed at recovery after FEP. To date, it is the most advanced online psychosocial intervention programme for early psychosis. The platform is based on social networking and therapeutic interventions targeting social functioning. Initial studies of this platform suggest that the application is feasible and safe (44,45). Most participants found the application useful and reported feeling more socially connected and more in control of their recovery (empowerment).

Horyzons is an online platform providing access to interactive psychosocial interventions targeting key factors in the recovery process. The Horyzons application offers psycho-educational content addressing topics such as understanding psychosis, identifying warning signs and preventing relapse. It incorporates cognitive behavioural therapy, positive psychology, mindfulness and meditation. It targets behavioural activation through action (“Do it”): for example, following a job search, users of the application find a behavioural suggestion encouraging them to “submit their CV to 10 different organisations”.The application also offers support from expert therapists and peer moderators. The therapists are registered mental health clinicians (clinical psychologists, social workers) and vocational specialists trained in Individual Placement and Support (IPS) with experience of working with young people with psychosis.

Finally, the application offers an online social network (“the Café”), where participants are encouraged to communicate with each other. The expert moderators are identifiable within the network. The network is also run by peer moderators called “peer-workers”: these are “trained young people with lived experience of mental illness”, whose role is to share useful content and encourage social interaction. Horyzons is moderated daily (2 hours per day on weekdays and 1 hour per day at the weekend).

The Australian team’s study of the Horyzons application (46) aims to assess whether digital intervention can be an effective strategy to extend the benefits of EIP treatment and promote social and vocational recovery beyond discharge from these programmes. The study compared 18 months’ use of the Horyzons smartphone application with treatment as usual. The patients were aged 16-27 years, with FEP and about to be discharged from their early intervention programme for transfer to treatment as usual (TAU). After discharge from two years on an early intervention programme, they were assigned at random to either TAU plus Horyzons or TAU alone for 18 months. TAU comprised various treatment options provided by medical or mental health services generally available to young people. Those with complex needs were referred to tertiary community mental health departments for adults, while those who had achieved a good level of recovery and clinical stability were referred to primary care departments. 86 participants (50.5%) were assigned at random to the Horyzons plus TAU group and 84 (49.5%) to the TAU alone group. Participants had an average age of 20.91 years.

Horyzons has been implemented in the United States and is assessed in the study by Ludwig et al. (12). The purpose of this study was to assess the acceptability and feasibility of Horyzons in the United States and to assess whether participation in this platform correlates with a reduction in the feeling of loneliness, and an improvement in social integration and the feeling of well-being. In the US study, the Horyzons platform did not include the employment and education support element, and the moderators were graduate students in clinical psychology, licensed clinical psychologists and clinical social workers. There were no peer moderators.

The Embrace programme targets social anxiety in the context of FEP (47). The application includes 12 online modules, as well as a discussion forum with expert and peer moderation. Each module includes clinical content targeting a CBT therapeutic goal specifically related to the management of social phobia, and is presented online in four formats: a brief psycho-educational description of each therapeutic concept; therapeutic comic books; behavioural experiments designed to address avoidance behaviours; and an interactive discussion feature in the form of an online social forum. The peer moderators are young people with lived experience of a mental health problem who have received peer support training. Their role is to provide support and encourage engagement (e.g. commenting on and liking messages). The status of the expert moderators is not specified. McEnery et al. (48) assessed the feasibility, acceptability, safety and preliminary effects of online intervention through Embrace. The study was a 12-week trial. The participants were young patients with FEP, who despite two years of specialist care and 18 months of online social support (participation in Horyzons) continued to experience social anxiety symptoms

Schlosser et al. demonstrated the feasibility and acceptability of Prime (personalised real-time intervention for motivational enhancement) (49), an online therapeutic intervention delivered via a mobile application developed at the University of California San Francisco (UCSF). Prime is designed to target motivation by setting goals to be achieved, with individualised CBT-based follow-up and coaching via a messaging service. The Prime application has three functions: goal monitoring with challenges (“My Goals”), messaging with individual and personalised motivational coaching (“Community”) and a social networking platform (“Moments”). In addition, the application includes a secured virtual community for interaction with other people sharing common interests or going through similar problems. The application helps participants work towards self-defined goals with the support of a virtual community of peers and coaches. Participants can select and document their progress on minor goals in four key areas: health/well-being, social relationships, creativity and productivity. Achievement of the goals is measured by the number of challenges completed in each area. They select these from a list of 36 items, which include goals such as “improving my relationship with my family” and “being more relaxed”. Suggestions include challenges such as “listen to relaxing music for five minutes” or “take a yoga class”, but participants can modify them or select other challenges that are easier to achieve. Participants receive automatic reminders of the challenges and must report when they are completed. They can share their achievements with their coach and the Prime community.

When a user signs up to the application, a motivational coach is assigned to them. These coaches are master’s level clinicians who use interventions drawn from CBT, behavioural activation, mindfulness and psycho-education to help participants overcome barriers to goal achievement. Coaches inform patients that they will be available to receive messages “most days” of the week, but that they will modify the frequency according to the patient’s preferences, clinical issues and overall progress towards the goals. As far as possible, messaging between coaches and participants is in real time. Participants can also ask to talk to the coaches by telephone or video call. Users can message each other directly and share moments from their daily lives with the Prime community.

The 2018 Prime study (16) was a 12-week randomised controlled trial to test the efficacy of the application, designed to improve motivation in the early stages of psychosis. The authors state: “Evidence suggests that negative symptoms, and lack of motivation in particular, play a major role in functional impairment in schizophrenia. Recent research in cognitive neuroscience has successfully characterised motivational deficits in schizophrenia.” The intervention has been designed to target the motivational system using social reinforcement. Participants were randomly assigned to the Prime application or TAU. Of the 43 participants recruited (mean age: 24.3 years), 22 were in the Prime group, 21 were in the TAU group, 5 dropped out and 6 did not complete follow-up. The trial used an exercise to assess three components of motivation: reward learning, anticipated pleasure and effort expenditure.

The Actissist application was also designed to help people with early psychosis. It is based on CBT and targets a number of areas in which people with early psychosis may report difficulties: going out; moving around; using cannabis; experiencing distress caused by psychotic symptoms such as paranoia and hearing voices (50).

The application contains initial active symptom monitoring (EMA) content with a series of structured self-assessment questions in question-and-answer format that focus on cognitive assessments, beliefs, emotions and associated behaviours. Depending on the assessment chosen, the Q&A is followed by suggestions (advice and tips) for CBT-based coping strategies in the form of a message. It also provides additional multimedia content with information and activities such as videos (personal recovery stories), a newsletter, links to external websites (TED conferences), fact sheets (on mood, anxiety, self-esteem), mindfulness and relaxation exercises and emergency contacts. It also features a chart showing historical variations in symptoms. The 2018 Actissist (51) study was a 12-week randomised controlled trial included 36 participants. This was the first study to compare an EMI application with a single EMA active control group in the “early psychosis” population. The study featured a 2:1 ratio for the Actissist plus TAU experimental group (n=24, mean age 20 years) compared with the ClinTouch plus TAU control group (n=12, mean age 18 years). Participants were aged over 16, in the first 3 years after their FEP, and on an EIP programme in England.
